# Formulation and evaluation of therapeutic antimicrobial citrus and Manuka honey creams with aloe vera, mint essential oil, and Indian costus

**DOI:** 10.1038/s41598-025-90072-6

**Published:** 2025-03-03

**Authors:** Marwa N. Ahmed, Omnia A. Elnasser, Sara A. Farghali, Ossama A. Ibrahim, Hala R. Ali, Olfat S. Barakat

**Affiliations:** 1 Department of Microbiology, Faculty of Agriculture, Cairo University, El-Gamaa Street, Giza, 12613 Egypt; 2 Computational Biology Department, Nile University, 6th of October City, Giza 12677 Egypt; 3 Department of Dairy sciences, Institute of Food Industries and Nutrition Research, National Research Centre, Dokki, Giza, 12622 Egypt; 4 Bacteriology Department, Animal Health Research Institute (AHRI), Agriculture Research Centre (ARC), Dokki, Giza, 12618 Egypt

**Keywords:** Antimicrobials, Applied microbiology, Biofilms, Pathogens

## Abstract

Honey has long been recognized for its antimicrobial properties, attributed to components such as polyphenols and biodefense proteins. Among honey types, Manuka honey, rich in methylglyoxal, and citrus honey, abundant in flavonoids and bioactive enzymes, exhibit potent antimicrobial activity. This study aims to enhance the antimicrobial and antibiofilm efficacy of Manuka and citrus honey by incorporating natural additives—aloe vera, Indian costus, and mint essential oil—into cream formulations. Two emulsion types, aerosil fumed silica-based and arabic gum-based, were prepared and optimized using sonication. The antimicrobial activity of these formulations was assessed against pathogenic bacteria, including *Bacillus cereus*, *Pseudomonas aeruginosa*, *Salmonella enterica* subsp. *enterica* serovar Typhimurium, Methicillin-resistant *Staphylococcus aureus*, *Listeria monocytogenes*, *Micrococcus luteus*,* Escherichia coli* O157:H7, and *Klebsiella pneumoniae*, as well as fungi such as *Candida albicans* and *Aspergillus niger*. The most effective formulations demonstrated inhibition zones of up to 28 mm against *B. cereus* and 24 mm against *S.* Typhimurium. Additionally, antibiofilm activity was evaluated using a 3D biofilm model, with formulations containing citrus honey and Indian costus or Manuka honey and aloe vera achieving biofilm reductions of 44.39% and 21.33%, respectively, against *P. aeruginosa* and MRSA. Furthermore, the composition of the citrus honey was analyzed using gas chromatography-mass spectrometry to identify the volatile and non-volatile compounds contributing to their antimicrobial properties. These findings suggest that honey-based formulations enhanced with natural additives hold significant potential for combating biofilm-associated infections.

**Supplementary Information:**

The online version contains supplementary material available at 10.1038/s41598-025-90072-6.

## Introduction

Antimicrobial resistance poses a significant clinical challenge, responsible for an estimated 700,000 deaths annually worldwide due to drug-resistant infections^1^. This number is expected to rise dramatically in the coming years. Most bacteria have developed resistance towards commercially available antibiotics due to the misuse of antibiotics^2^. A systematic analysis of the Global Burden of Disease Study revealed that in 2019, 33 bacterial pathogens were associated with significant global mortality, highlighting the urgent need for innovative antimicrobial strategies^3^. Bacteria predominantly exist in their natural habitats in the form of biofilms to withstand extreme conditions^4^. Biofilms, which are a group of cells attached together and encased in protective extracellular matrices, are a major contributor to chronic wound infections^5^. These structures shield bacteria from the immune system and antibiotic treatments, allowing infections to persist and become resistant to antibiotic therapies^5^. Chronic wounds, such as diabetic foot ulcers and pressure sores, are often the result of biofilm formation, making effective treatments for these infections essential^6^. Therefore, research into alternative natural antimicrobial agents and effective delivery systems is crucial in combating multidrug-resistant pathogens.

Honey has long been recognized for its therapeutic potential and remains a vital natural remedy for various diseases^7^. Honey exerts antimicrobial properties, primarily due to its inherent components such as methylglyoxal (MGO), polyphenols, and bee defensin-1, which enable it to combat infections^8^. However, not all types of honey exhibit the same antimicrobial effects. For instance, Manuka honey pose a remarkable antimicrobial effect due to its high content of methylglyoxal, pinobanksin, and glyoxal, making it particularly effective in wound care and microbial infection control^9^. Manuka honey’s low pH (3.5–4.5) enhances its bactericidal properties by stimulating macrophage activity, reducing protease action, and promoting oxygenation in chronic wounds, which are often associated with biofilm formation^10^.

Manuka honey’s unique antimicrobial profile, particularly its effectiveness against biofilm-associated pathogens such as *Staphylococcus aureus*, *Streptococcus pyogenes*, *Proteus mirabilis*, *Enterobacter cloacae*, *Pseudomonas aeruginosa*, *Listeria moncytogenes*, and *Escherichia coli*, is largely attributed to its non-peroxide activity, driven by methylglyoxal^8–10^. Citrus honey, another type of honey, derived from the nectar of citrus blossoms, contains flavonoids, polyphenols, hydrogen peroxide^11^, and volatile compounds like limonene and pinene^12^. These components contribute to its antimicrobial properties, although its antimicrobial activity primarily relies on hydrogen peroxide production for its antimicrobial activity. Citrus honey’s lower MGO content compared to Manuka honey makes it less effective against certain pathogens. However, its phenolic compounds and volatile oils provide a broader antioxidant and mild antimicrobial profile^13^. Previous studies have shown that citrus honey inhibits the growth of bacterial pathogens such as *S. aureus*, *E. coli*, *P. aeruginosa*, and *Klebsiella pneumoniae*^14^.

Several natural plant-based compounds, such as aloe vera, Indian costus (*Saussurea costus*), arabic gum, and mint essential oil, pose strong antimicrobial and antioxidant effects^15–17^. Aloe vera, with its antibacterial, anti-inflammatory, and immunomodulatory properties, has been shown to effectively combat a range of Gram-positive and Gram-negative bacteria, including *K. pneumoniae*, *S. aureus*, *P. aeruginosa*, and *E. coli*^18,19^. The active components of aloe vera, such as saponins and anthraquinones, contribute to its antimicrobial capabilities. Indian costus has also been widely studied for its broad-spectrum antimicrobial activities, making it a promising natural alternative to synthetic preservatives^20^. Similarly, *Saussurea* *costus* has demonstrated broad antimicrobial activity against a wide range of pathogenic microorganisms^21,22^. Mint essential oil, commonly used in the pharmaceutical and cosmetic industries, also exhibits antimicrobial activity against both Gram-positive and Gram-negative bacteria^23^. Additionally, it has demonstrated antiviral and antifungal properties, enhancing its versatility as a natural remedy^23^. Arabic gum, derived from the resin of *Acacia senegal* and *Acacia seyal*, exhibits notable antimicrobial activity due to its high polysaccharide content, which can inhibit the growth of various bacteria and fungi, making it a valuable compound in both food preservation and pharmaceutical applications^24^. Recent advancements have proposed innovative therapeutic strategies targeting intracellular multidrug-resistant bacteria, emphasizing the potential of combining novel delivery systems with natural antimicrobial agents^25^.

Therefore, the current study aims to develop various therapeutic cream formulations incorporating both Manuka and citrus honey, enhanced with natural compound additives. These formulations were then evaluated for their antimicrobial efficacy against a range of pathogenic microorganisms. Additionally, this study assessed the antibiofilm activity of these formulations against mature biofilms, which were formed by common skin pathogens. A 3D-biofilm model was utilized to simulate in vivo conditions, specifically targeting biofilms associated with chronic wounds. Furthermore, the composition of the citrus honey was analyzed using gas chromatography-mass spectrometry (GC-MS) to identify the volatile and non-volatile compounds responsible for the antimicrobial and antibiofilm properties of the formulations.

## Materials and methods

### Emulsion preparation and cream formulation

**Fig. 1:**
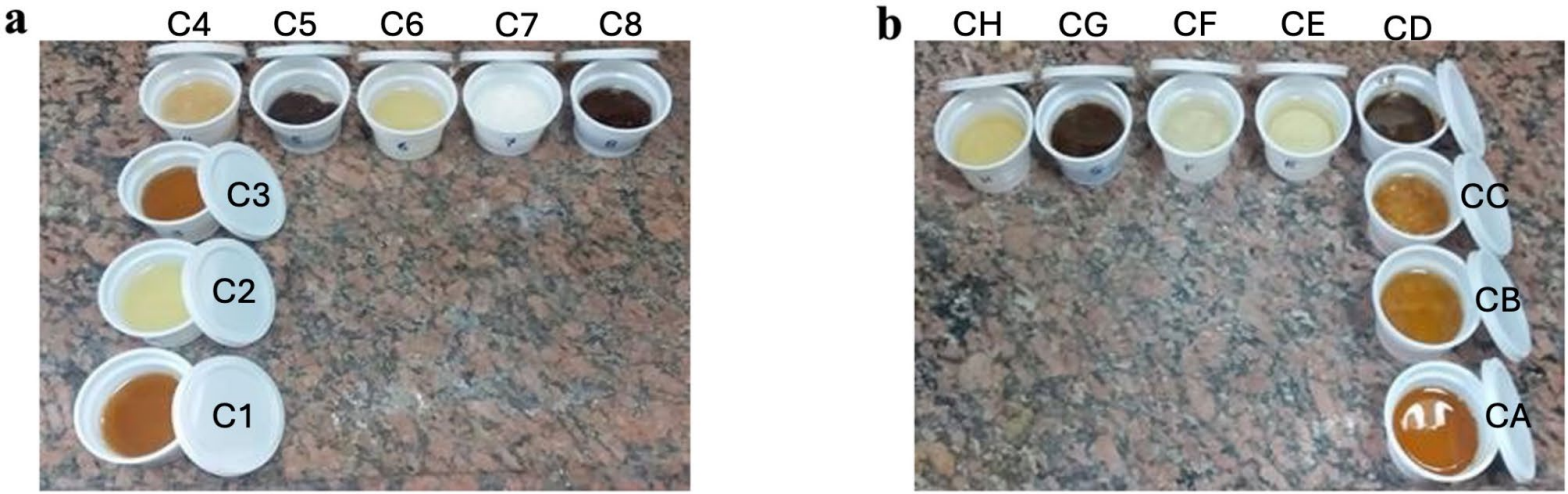
Different cream formulations based on Manuka or citrus honey (**a**) and silica emulsions or (**b**) AG emulsions.

Different creams were formulated using either Manuka or citrus honey (supplied from the local market and used as delivered without any further purification or modifications), combined with silica or arabic gum (AG) as thickeners. Aloe vera gel (AVG), Indian costus (IC), and mint essential oil (MEO) were incorporated as antimicrobial agents in various formulations, as detailed in Table 1. Two types of emulsions were created using either Manuka or citrus honey in a 1:1 ratio: (a) The first emulsion was prepared with aerosil fumed silica, paraffin oil, and Tween 20 in a 1:4:4 ratio (Fig. 1a). (b) The second emulsion was made with AG at 1% (w/v) in glycerol (Fig. 1b). Each emulsion underwent sonication for 5 min using a 400-watt ultrasonic micro-tip probe (Ultrasonic Get 750). Following the addition of Manuka or citrus honey along with other additives (Table 1), the sonication was continued for an additional 5 min to ensure proper homogenization. All emulsions were prepared at room temperature.

**Table 1:** The Formulation of different creams based on Manuka and citrus honey emulsions.

Prepared cream samples	Additives in first emulsion	Prepared cream samples	Additives in second emulsion
Cream 1 (C1)	Silica emulsion and Manuka honey (1:1)	Cream A (CA)	AG emulsion and Manuka honey (1:1)
Cream 2 (C2)	Silica emulsion (25 mL), and Manuka honey (22.5 g) contains MEO (2.5 mL)	Cream B (CB)	AG emulsion (25 mL) and Manuka honey (22.5 g) contains MEO (2.5 mL)
Cream 3 (C3)	Silica emulsion (25 mL) and Manuka honey (12.5 g) contains AVG (12.5 g)	Cream C (CC)	AG emulsion (25 mL) and Manuka honey (12.5 g) contains AVG (12.5 g)
Cream 4 (C4)	Silica emulsion (25 mL) and Manuka honey (21 g) contains IC (4 g)	Cream D (CD)	AG emulsion (25 mL) and Manuka honey (21 g) contains AVG (4 g)
Cream 5 (C5)	Silica emulsion and citrus honey (1:1)	Cream E (CE)	AG emulsion and citrus honey (1:1)
Cream 6 (C6)	Silica emulsion (25 mL) and citrus honey (22.5 g) contains MEO (2.5 mL)	Cream F (CF)	AG emulsion (25 mL) and citrus honey (22.5 g) contains MEO (2.5 mL)
Cream 7 (C7)	Silica emulsion (25 mL) and citrus honey (12.5 g) contains AVG (12.5 g)	Cream G (CG)	AG emulsion (25 mL) and citrus honey (12.5 g) contains AVG (12.5 g)
Cream 8 (C8)	Silica emulsion (25 mL) and citrus honey (21 g) contains IC (4 g)	Cream H (CH)	AG emulsion (25 mL) and citrus honey (21 g) contains AVG (4 g)

First Emulsion: prepared using aerosil fumed silica, paraffin oil, and Tween 20 in the ratio of 1:4:4, respectively. Second emulsion: prepared from AG at the level of 1% (w/v) in glycerol. AG: arabic gum.

AVG aloe vera gel, IC Indian Costus, MEO mint essential oil.

### **Bacterial strains**

The indicator pathogenic bacteria used in this study were purchased from American-type culture collection (ATCC). These pathogens include *Bacillus cereus* ATCC 33,018, *Escherichia coli* O157:H7 ATCC 700,728, *Klebsiella pneumoniae* ATCC 13,883, *Listeria monocytogenes* ATCC 19,115, *Micrococcus luteus* ATCC 10,240, Methicillin-resistant *Staphylococcus aureus* (MRSA) ATCC 43,300, *Pseudomonas aeruginosa* ATCC 35,032, *Staphylococcus aureus* ATCC 25,923, *Salmonella enterica* subsp. *enterica* serovar Typhimurium ATCC 14,028, *Candida albicans* ATCC 10,231, and *Aspergillus niger* NRRL 326. All pathogenic bacterial strains were cultured and propagated on a Luria Bertani (LB) broth medium under aerobic conditions at 37 °C with the exception of  *B. cereus*, which was incubated at 30 °C. Fungal strains were cultured and propagated on a Sabouraud dextrose broth (SDB) medium under aerobic conditions, with *A. niger* incubated at 30 °C and *C. albicans* at 37 °C. These conditions were maintained to support the specific growth requirements of each microorganism.

### **Agar well diffusion assay**

The agar well diffusion method was employed in this study to assess the antimicrobial activity. Mueller-Hinton (MH) agar plates were employed for bacterial growth, while Sabouraud dextrose agar (SDA) was used for fungal growth. To ensure uniform microbial growth, the agar plates were inoculated by evenly spreading microbial suspensions across the entire surface. Wells, typically 6 to 8 mm in diameter, were aseptically punched into the agar using a sterile cork borer. Each well was carefully filled with 100 µL of the antimicrobial cream or control samples. Positive controls were included for comparative purposes, with novobiocin used for Gram-positive bacteria and polymyxin B for Gram-negative bacteria. For the negative control, distilled water was used. The plates inoculated with bacterial pathogens were incubated under aerobic conditions at 37 °C for 24 h, except for *B. cereus*, which was incubated at 30 °C for the same duration. Plates inoculated with *A. niger* were incubated at 30 °C for seven days, while those inoculated with *C. albicans* were incubated at 37 °C for 48 h. Following the incubation peroid, the diameter of inhibition zones was measured.

### Development of 2D biofilm

Both *P. aeruginosa* and MRSA strains were cultured overnight at 37 °C with shaking in LB broth. Following incubation, 100 µL of the bacterial suspension was transferred into each well of a 96-well microtiter plate. The plates were sealed with Parafilm and placed in a sealed container along with two 200 mL beakers of water to maintain humidity. The plates were incubated for 72 h at 37 °C. After incubation, planktonic cells were discarded by gently inverting the 96-well plate over a waste container. The biofilm was then carefully scraped and collected using a pipette^26^.

### Alginate beads synthesis

Biofilm beads were synthesized based on a previously published method with slight modifications^27^. A 4% (w/v) solution of sodium alginate (Sigma Aldrich, St. Louis, MO, USA) was prepared, and 250 µL of freshly collected biofilm bacteria (as described above) was mixed with 1.5 mL of the alginate solution. The biofilm-alginate mixture was then dispensed dropwise into a crosslinking solution composed of 1.5% CaCl₂ in 13 mM HEPES buffer (Sigma Aldrich) using a sterile syringe fitted with a 25 G needle, while gently swirling the beaker to ensure even crosslinking. The beads were allowed to crosslink at room temperature for 20 min. After crosslinking, the solution was removed, and the beads were washed twice with sterile 13 mM HEPES buffer. The biofilm beads were then distributed into 96-well plates containing LB broth, with one bead per well. To test the stability of the beads, they were incubated in LB broth for 2 weeks, with the medium being replaced every 3 days.

### Construction of 3D biofilm model

The 3D biofilm model was developed by embedding biofilm beads between two layers of alginate matrix. To begin, 150 µL of 4% alginate solution was applied as the first matrix layer onto ThinCert^®^ Cell Culture inserts (1.0 μm pore diameter, transparent, sterile) designed for 24-well plates (Greiner Bio One, Gloucestershire, UK). The alginate layer was cross-linked by exposure to a CaCl₂/HEPES buffer solution for 5 min, followed by two washes with sterile 13 mM HEPES buffer. Two 5-day-old biofilm beads were then placed on top of the first alginate layer. A second layer of 150 µL of 4% alginate was applied to cover the beads, forming the complete matrix. This resulted in the biofilm beads being encased between two layers of alginate matrix, creating the 3D biofilm model^26^.

### Evaluating the constructed 3D biofilm model

The constructed 3D biofilm model was treated with four cream formulations (C5, C7, C8, and CB ) at a ratio of 1:1 cream to LB broth for a period of 3 days. After incubation, the alginate matrix from both treated and untreated control groups was carefully removed from the Boyden chamber inserts using a sterile loop. The removed matrix was then un-crosslinked by incubating it in 1 mL of 33 mM trisodium citrate (Fisher Scientific, Hampton, NH, USA) for 20 min. Following un-crosslinking, the bacterial content within the matrix was quantified by performing 10-fold serial dilutions and plating on LB agar^26^.

### GC-MS analysis of citrus honey

The volatile and non-volatile compounds in citrus honey were analyzed using a GC-MS/MS triple quadrupole system (Agilent Technologies 7890B), following the methodology described by Alissandrakis et al. (2005) and Wang et al. (2025)^28,29^. For the extraction of volatile compounds, solid-phase microextraction (SPME) was employed. Briefly, 1–2 g of citrus honey were placed in a vial, to which 10 mL of deionized water was added to facilitate dilution. Sodium chloride was then added to the vial to saturation, enhancing the volatilization of the compounds. The sealed vial was subsequently heated to a temperature range of 40–60 °C for a period of 30–60 min to encourage the release of volatiles into the headspace. During this process, an SPME fiber (PDMS or DVB/CAR/PDMS) was exposed to the headspace for a specified duration to absorb the released volatile compounds.

For the extraction of non-volatile compounds, 5 g of citrus honey were diluted with 10 mL of distilled water. This mixture was then subjected to liquid-liquid extraction using 10 mL of an organic solvent, such as dichloromethane or ethyl acetate. After phase separation, the organic phase was carefully collected, and the solvent was removed under reduced pressure. The resulting extract was reconstituted in a GC-compatible solvent (methanol) before conducting analysis by GC-MS triple quadrupole system. This dual extraction approach enabled the comprehensive profiling and quantification of both volatile and non-volatile constituents within the citrus honey.

### Statistical analysis

Graphs and statistical analyses were conducted using R software (version 3.2.5). Depending on the experimental design, one-way or two-way analysis of variance (ANOVA) was applied to evaluate the significance of comparisons. Tukey’s Honestly Significant Difference (HSD) test was used for pairwise comparisons to test significant main effects or interactions. A p-value of ≤ 0.05 was considered statistically significant.

## Results

### Antimicrobial susceptibility analysis of cream formulations against pathogenic bacteria and fungi

**Fig. 2:**
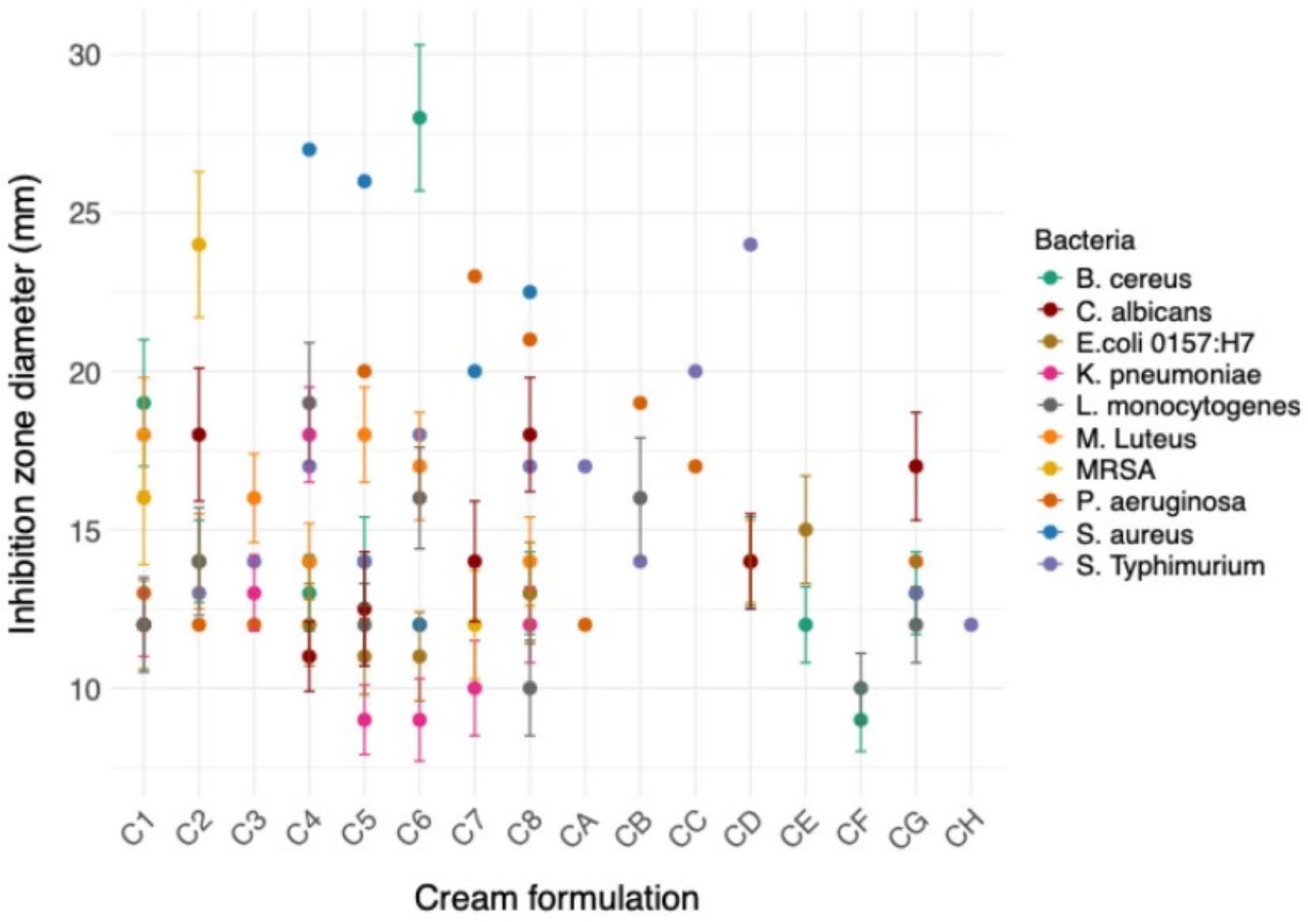
The antimicrobial effect of various cream formulations prepared in this study against different pathogenic bacterial and fungal strains using well diffusion assay. The values represent the means of three independent replicates of the inhibition zone diameter (mm).The error bars represent the standared error of the mean (SEM).

The antimicrobial sensitivity of different bacteria and fungi towards to the prepared cream formulations was assessed using well diffusion assay. A larger inhibition zone corresponds to greater antimicrobial efficacy of the formulation against the tested microorganism. Interestingly, cream formulations based on citrus honey and silica emulsion showed a higher antimicrobial efficacy compared to those based on Manuka honey and AG against the tested pathogenic bacteria and fungi (Fig. 2 and Table S1). The pathogenic bacteria being tested in this study were more affected by various cream formulations compared to the tested fungi. C8 formulation exhibited the highest antimicrobial activity against a wide range of pathogenic bacteria compared to other cream formulations.

For *B. cereus*, several cream formulations exhibited antimicrobial activity, with cream formulation C6 showing the highest inhibition zone of 28 ± 2 mm, indicating strong efficacy against this Gram-positive bacterium. Other formulations such as C1, C2, C4, C5, CD, and CG displayed moderate inhibition zones ranging from 13 to 19 mm, while some formulations did not exhibit any inhibitory effects. In the case of *P. aeruginosa*, a Gram-negative bacterium known for its resistance to many antibiotics, the most potent formulation was C7, with an inhibition zone of 23 ± 2.1 mm. Other formulations, such as C5, C8, and CB, also showed a high efficacy with inhibition zones ranging from 19 to 21 mm. Formulations C1 to C4 and C6 generally displayed moderate activity, while formulations CD, CE, and CF showed no activity.

For *S.* Typhimurium, formulation CD was the most effective, with an inhibition zone of 24 ± 1.9 mm, followed by formulation CC (20 ± 1.4 mm). The activity of the cream formulations against *K. pneumoniae* was relatively limited, with formulation C4 demonstrating the highest inhibition zone of 18 ± 1.9 mm. Formulations C1, C3, C5, C6, and C7 showed minimal to moderate activity, while the other formulations did not exhibit any antimicrobial effects. For *M. luteus*, formulations C1, C5, and C6 were the most effective, with inhibition zones of 18 ± 1.4 mm,18 ± 1.5 mm, and 17 ± 1.4 mm, respectively. The formulation C2 was the most potent against MRSA, with an inhibition zone of 24 ± 1.9 mm, indicating strong antimicrobial activity. Formulation C1 also showed significant activity with an inhibition zone of 16 ± 1.6 mm, while other formulations displayed minimal or no activity. For *E. coli* O157:H7 formulation CE was the most effective with a 15 ± 1.5 mm inhibition zone. Formulations C1, C4, C5, C6, and C8 displayed moderate activity with inhibition zones ranging from 11 to 13 mm, while the remaining formulations had no significant activity. For *L. monocytogenes*, formulation C4 was the most effective, with an inhibition zone of 19 ± 1.8 mm, followed by formulations C6 and C2, each with inhibition zones ranging from 14 to 16 mm. Formulations C4 and C5 showed the highest antimicrobial activity against *S. aureus*, with inhibition zones of 27 ± 1.8 mm and 26 ± 2 mm, respectively. Formulations C7 and C8 also exhibited significant activity with inhibition zones of 20 ± 2 mm and 22.5 ± 2.1 mm, respectively.

For *C. albicans* yeast, formulations C2, C8, and CG were the most effective with inhibition zones ranging from 17 to 18 mm. The remaining formulations either had moderate or no significant activity. *A. niger* was not affected by any of the tested formulations, as indicated by the absence of inhibition zones across all formulations for this fungus.

### The antibiofilm activity of cream formulations against pathogenic bacteria and fungi using 3D biofilm model

**Fig. 3:**
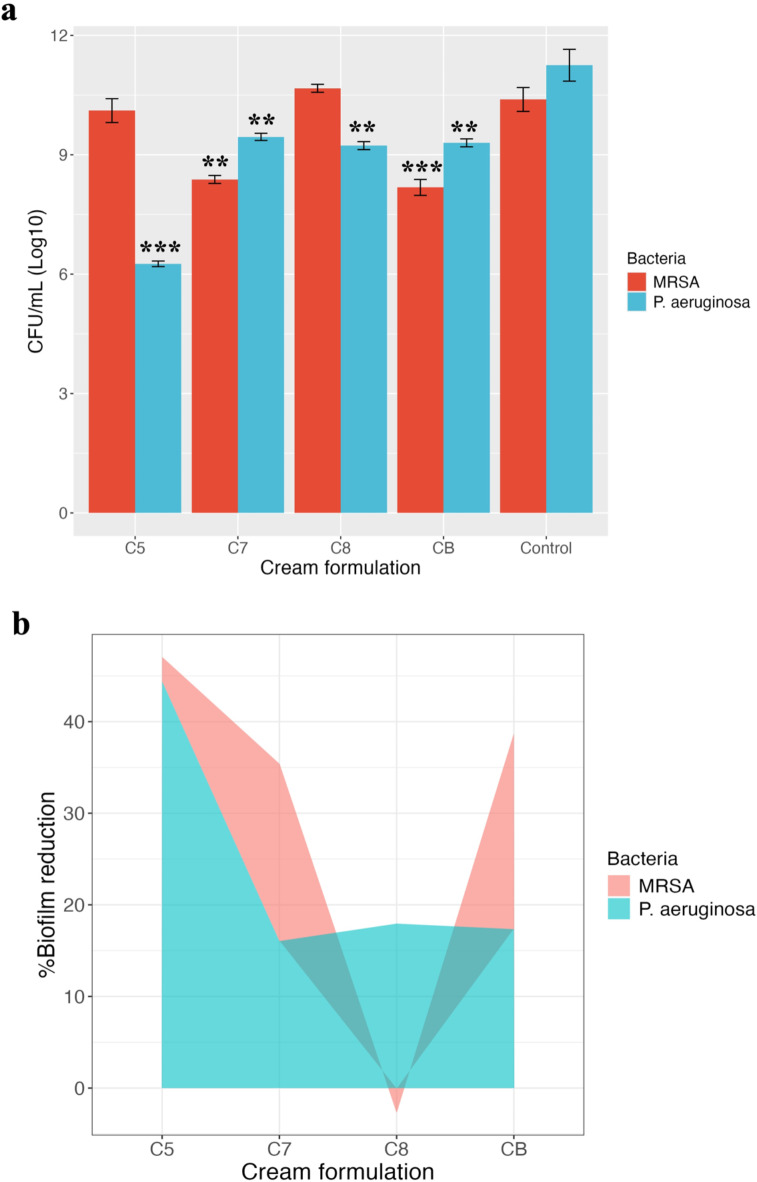
The antibiofilm activity of different cream formulations (C5, C7, C8, and CB) against MRSA and *P. aeruginosa* biofilms grown in 3D biofilm model. (**a**) The antibiofilm activity expressed as CFU/mL (Log10). Error bars represent SEM of five independent replicates. Statistical significance is indicated: *P* < 0.01 (**), *P* < 0.001 (***), compared to the control group. (**b**) Percentage biofilm reduction of MRSA and *P. aeruginosa* upon treatment with the cream formulations. Data are presented as means of five independent replicates with shaded regions representing SEM.

The effects of the cream formulations C7, C8, C5, and CB on dispersing and killing cells within well-established biofilms formed by MRSA and *P. aeruginosa* were evaluated using a 3D biofilm model to simulate the in vivo biofilm formation. The results are presented as log10 CFU/ml, representing the bacterial load under different treatment conditions, including untreated controls and treatment with the cream formulations at a ratio of 1:1 cream to LB broth (Fig. 3).

At the 1:1 ratio, the cream formulations demonstrated varying effects on biofilm dispersal and bacterial killing. For MRSA, the C7 formulation, composed of citrus honey, silica emulsion, and IC, reduced the bacterial count to a log10 value of 8.38 ± 0.1, indicating a moderate effect on biofilm dispersal and bacterial killing. In contrast, the C8 formulation, which includes citrus honey, silica emulsion, and AVG, and the C5 formulation, containing only citrus honey and silica emulsion, did not significantly impact the bacterial count, showing no clear effect on MRSA biofilm disruption. Interestingly, the CB formulation, a Manuka honey cream with AV as an additive, resulted in a significant reduction in the bacterial load, bringing the log10 CFU/ml value to 8.18 ± 0.2, suggesting that this formulation has potent biofilm-disrupting and killing properties against MRSA (Fig. 3a).

In the case of *P. aeruginosa*, all cream formulations at the 1:1 ratio effectively reduced the bacterial load, indicating a significant disruption of the biofilm. The C5 formulation was particularly potent, reducing the bacterial count to 6.26 ± 0.07 log10 CFU/ml, suggesting a strong effect on biofilm disruption and bacterial killing (Fig. 3a).

Figure 3b illustrates the percentage of biofilm reduction and killing for MRSA and *P. aeruginosa* in response to the cream formulations at the 1:1 ratio. For MRSA, both the C7 and CB formulations achieved significant reductions compared to other formulations (*P* < 0.05), with biofilm reduction and killing rates of 19.36% and 21.33%, respectively. In the case of *P. aeruginosa*, the C5 formulation demonstrated the highest signifcant efficacy (*P* < 0.01), with a biofilm reduction of 44.39%.

### GC-MS analysis of citrus honey

Citrus honey was subjected to GC-MS analysis to determine the compounds contributing to its potent antimicrobial activity. The GC-MS analysis of citrus honey identified four major compounds contributing to its chemical profile.(Table 2). Each compound was characterized by its retention time, molecular weight, molecular formula, and intensity. The most abundant compound identified was 5-Hydroxymethylfurfural (HMF), detected at a retention time of 23.278 min.

**Table 2:** The most abundant compounds in citrus honey identifed using GC-MS with their retention times, molecular weights, molecular formulas, and intensities.

Compound	Retention Time (min)	Molecular Weight (g/mol)	Molecular Formula	Intensity
Methyl 2-furoate	6.362	112.10	C_6_H_6_O_3_	620,780.63
1,3,5-Benzenetriol	15.154	126.11	C_6_H_6_O_3_	348,000.27
5-Hydroxymethylfurfural	23.278	126.11	C_6_H_6_O_3_	2,906,299.25
n-Hexadecanoic acid	42.059	256.42	C_16_H_32_O_2_	511,097.69

## Discussion

This study investigated the preparation of diverse cream formulations incorporating Manuka and citrus honey, supplemented with either AVG, IC, or MEO, using AG or silica emulsion as thickeners. In the silica-based emulsions, silica acts as a stabilizer, creating a stable oil-in-water emulsion due to its hydrophilic-lipophilic balance properties. Similarly, in AG-based emulsions, AG serves as a natural emulsifier, leveraging its amphiphilic nature to stabilize the emulsion. The aqueous phase in these formulations is provided by the honey and any hydrophilic additives, while paraffin oil serves as the oil phase^30,31^. These multiple formulations aim to evaluate how the inclusion of different natural additives such as IC, MEO, and AVG in conjunction with Manuka or citrus honey impacts the antimicrobial and antibiofilm activities of the creams.

The antimicrobial susceptibility analysis of these formulations demonstrated varied efficacy against a range of pathogenic bacteria and fungi, emphasizing the influence of formulation composition on antimicrobial activity. Generally, formulations based on citrus honey and silica emulsion exhibited superior antimicrobial activity compared to those based on Manuka honey and AG. This difference highlights the synergistic interaction between citrus honey, silica, and other additives, enhancing the antimicrobial properties of these formulations.

The formulations demonstrated broad-spectrum antimicrobial activity against Gram-positive and Gram-negative bacteria. Specifically, *B. cereus*, a Gram-positive bacterium, was highly susceptible to the C6 formulation, which exhibited the highest inhibition zone (28 ± 2 mm). This enhanced activity may be attributed to the synergistic effect of citrus honey and silica emulsion, which likely disrupts bacterial cell walls. The synergestic effect of the combination between citrus honey and other componds such as antibiotics have been shown previously^32^. Other formulations (C1, C2, C4, C5, CD, and CG) exhibited moderate activity, further highlighting the antimicrobial potential of citrus-honey-based creams.

*P. aeruginosa* is a challenging Gram-negative pathogen known for its inherent antibiotic resistance. In this study, the C7 formulation exhibited significant efficacy against *P. aeruginosa*, with an inhibition zone of 23 ± 2.1 mm. This notable activity may be attributed to the synergistic effects of citrus honey, silica emulsion, and IC, which likely enhance biofilm disruption and cell membrane penetration. Previous research has demonstrated that honey possesses broad-spectrum antimicrobial properties, including activity against *P. aeruginosa*, due to its ability to inhibit quorum sensing and disrupt biofilms^33^. Additionally, studies on silver–silica nanocomposites have shown their effectiveness in compromising the cell wall integrity and metabolism of *P. aeruginosa*, leading to increased susceptibility^34^. The incorporation of IC, known for its antimicrobial properties, may further contribute to the observed efficacy. Formulations C5, C8, and CB also displayed considerable activity against *P. aeruginosa*, indicating the potential of these combinations. Further investigation into their specific modes of action is warranted to fully understand their therapeutic potential.

Against *S.* Typhimurium, formulation CD was the most effective (24 ± 1.9 mm). This suggests that the combination of Manuka honey and AG in CD formulation may offer targeted antimicrobial effects. However, the overall efficacy of this formulation was not consistently superior across all tested bacteria. Limited activity against *K. pneumoniae* was noted, with only formulation C4 showing a notable inhibition zone (18 ± 1.9 mm). The efficacy against MRSA was particularly promising, with formulation C2 exhibiting the highest activity (24 ± 1.9 mm). The efficacy of the formulations against MRSA was particularly promising. Formulation C2 demonstrated the highest activity (24 ± 1.9 mm), highlighting its potential for treating MRSA infections, an increasing concern due to antibiotic resistance^35^. Formulation C1 (16 ± 1.6 mm) also showed moderate activity. The antimicrobial properties of diiferent Pakistani honeys were evaluted against *S.* Typhimurium showing high efficacy^36^. Additionally, the antimicrobial properties of Manuka honey have been well-documented, particularly against Gram-positive bacteria such as MRSA. Studies have shown that Manuka honey exhibits significant antibacterial activity against MRSA strains, making it a potential alternative treatment for antibiotic-resistant infections^37^. These findings highlight the potential of citrus honey and silica emulsion-based creams as alternative therapies for antibiotic-resistant infections.

The antifungal activity of the formulations was less pronounced compared to their antibacterial effects. For *C. albicans*, formulations C2, C8, and CG showed inhibition zones between 17 and 18 mm, indicating moderate antifungal effects. These results suggest that while the formulations possess some antifungal properties, further optimization may be required to enhance their effectiveness against fungal pathogens. On the other hand, *A. niger* exhibited resistance to all tested formulations, likely due to the inherent structural robustness of its spores and hyphae. The tough outer layers of *A. niger*, including its thick spore wall and the production of biofilms, pose significant challenges for antimicrobial agents, which often require enhanced potency or specialized mechanisms to penetrate and disrupt these defenses^38^.

The differences in antimicrobial activity are influenced by the structural complexity of microbial cell walls and membranes, intrinsic resistance mechanisms, and the specific bioactive components of each formulation. The high antimicrobial efficacy of citrus-honey-based formulations can be attributed to flavonoids and phenolic compounds, which target microbial cell walls and membranes^39^. In contrast, Manuka honey, rich in MGO, demonstrated selective activity depending on the microbial target. Silica emulsion enhanced antimicrobial efficacy by providing a stable delivery matrix for active compounds. Essential oils, such as those in formulations C2 and C6, disrupted microbial membranes through interactions with phospholipids, causing leakage of cellular contents^40^. Gram-positive bacteria, with their thick peptidoglycan layers, were generally more susceptible to these formulations, while Gram-negative bacteria, with their outer lipopolysaccharide membranes, exhibited reduced susceptibility. Formulations like C7 overcame these defenses through components such as MEO, which disrupts lipid bilayers.

The efficacy of the formulations in disrupting biofilms and killing bacterial cells within the biofilm was assessed using a 3D biofilm model, which mimics the in *vivo* environment. Biofilms pose a significant barrier to the action of antimicrobial agents due to their ability to limit drug penetration, reduce metabolic activity of embedded cells, and induce the expression of resistance mechanisms^41^. As such, breaking down biofilms is a crucial challenge in the treatment of chronic infections, especially those caused by multidrug-resistant bacteria like MRSA^42^.

In this study, formulations C7 and CB demonstrated moderate biofilm disruption in MRSA. At a 1:1 cream-to-LB broth ratio, both formulations achieved log10 reductions of 8.38 ± 0.1 and 8.18 ± 0.2 CFU/ml, respectively. These reductions can be attributed to the unique combinations and specific ratios of the components in C7 and CB creams, which may synergistically enhance their efficacy. Although other formulations share individual ingredients with C7 or CB, the precise balance of citrus honey and AVG in C7, as well as Manuka honey and MEO in CB, appears to create an optimal microenvironment that facilitates biofilm penetration and disruption. These specific ratios likely optimize the antimicrobial properties of the components, enhancing their efficacy. Additionally, the synergistic interactions between the ingredients in C7 and CB may play a crucial role. For instance, AVG in C7 might enhance the penetration of active compounds through the biofilm matrix, while citrus honey contributes potent antimicrobial activity. Similarly, the combination of Manuka honey and MEO in CB provides broad-spectrum antimicrobial effects and disrupts the biofilm architecture effectively. Furthermore, the emulsion base (silica in C7 and AG in CB) may influence the stability, delivery, and prolonged release of active ingredients, ensuring targeted and effective action against MRSA biofilms. This indicates the importance of formulation optimization, including precise ingredient selection and ratio balancing, in enhancing the efficacy of biofilm-targeting antimicrobial therapies. The potential of honey and plant essential oils to reduce biofilm formation and promote the dispersal of biofilms have been demonstrated previously^43,44^.

For *P. aeruginosa*, a pathogen known for its ability to form resilient biofilms that protect against immune responses and antibiotics^45^, all formulations showed significant biofilm disruption. The C5 formulation exhibited the most substantial reduction in biofilm viability, achieving a log10 reduction of 6.26 ± 0.07 CFU/ml. This finding suggests that C5 may have superior efficacy in penetrating and disrupting the biofilm matrix, a challenge due to the biofilm’s extracellular polymeric substance (EPS), which acts as a physical barrier to antimicrobial agents. C5 consists of a 1:1 ratio of silica emulsion to citrus honey, creating a highly concentrated formulation with a balance that may enhance the antimicrobial activity of the honey while maintaining an optimal consistency for biofilm penetration.

Biofilm-associated infections caused by *P. aeruginosa* are particularly problematic in clinical settings, as this pathogen is known to be resistant to many antibiotics, including those that target planktonic cells^46^. Honey has been shown previously to effectively reduce and eliminate biofilms formed by *P. aeruginosa*^47^. This disruption is particularly important in clinical settings, where *P. aeruginosa* biofilms contribute to persistent infections, especially in cystic fibrosis patients and those with implant-related infections^48^. These results emphasize the potential of formulation C5 as a promising therapeutic candidate for treating *P. aeruginosa* biofilm-related infections by disrupting the biofilm architecture and enhancing the penetration of antimicrobial agents.

Given the high antimicrobial activity observed in citrus-honey-based formulations, we conducted a comprehensive GC-MS analysis of citrus honey to identify the bioactive compound. The analysis revealed four major constituents, with 5- HMF emerging as the most abundant compound. HMF is a furan derivative with an aldehyde and a hydroxymethyl group, formed from reducing sugars. HMF is a well-established bioactive compound with robust antimicrobial and antioxidant properties^49^. Alongside HMF, the analysis identified methyl 2-furoate (an ester of furoic acid), 1,3,5-benzenetriol (phenolic compound), and n-hexadecanoic acid (saturated fatty acid), each of which has been implicated in antimicrobial activity. These compounds exert their effects through diverse mechanisms, including microbial membrane disruption, metabolic pathway interference, and oxidative stress induction^50^.

## Conclusion

This study demonstrates the significant antimicrobial and biofilm-disrupting potential of citrus-honey-based formulations, particularly those incorporating natural additives such as AVG, IC, and MEO. The formulations showed broad-spectrum antimicrobial activity, with citrus honey-based creams, especially C5, C6, and C7, proving effective against key pathogens such as *B. cereus* and *P. aeruginosa*, while Manuka-honey-based formulations, particularly C2, C4, and CD, were more effective against MRSA and *S.* Typhimurium. Additionally, moderate antifungal activity was observed against *C. albicans*. Biofilm disruption was demonstrated with significant reductions in *MRSA* biofilm viability in formulations C7 and CB. C5 exhibited the most substantial reduction in *P. aeruginosa* biofilm viability, demonstrating its potential in disrupting biofilm-associated infections caused by this resilient pathogen. The GC-MS analysis of citrus honey identified four major bioactive compounds, including 5-HMF, methyl 2-furoate, 1,3,5-benzenetriol, and n-hexadecanoic acid, all of which contribute to the antimicrobial and antioxidant properties of the formulations. These findings suggest that citrus-honey-based creams, particularly when optimized with specific bioactive compounds, show promise as effective therapies for bacterial infections, including antibiotic-resistant strains.

Future research could significantly expand upon these findings by further investigating the underlying mechanisms driving the antimicrobial and biofilm-disrupting effects of the individual compounds identified through GC-MS analysis, with particular emphasis on the pivotal role of 5-HMF. A deeper understanding of how 5-HMF interacts with microbial cell structures, metabolic processes, and resistance mechanisms could unlock new avenues for its application in antimicrobial therapies. Furthermore, exploring the synergistic effects of 5-HMF in combination with other bioactive compounds, such as methyl 2-furoate and n-hexadecanoic acid, could provide valuable insights into their collective impact on biofilm disruption and microbial susceptibility. This provides promising avenues to develop novel, natural-based therapies for the management of persistent infections in chronic wounds, potentially addressing the growing concern of antibiotic resistance.

## Electronic supplementary material

Below is the link to the electronic supplementary material.

Supplementary Material 1

## Data Availability

The datasets generated during and/or analyzed during the current study are available from the corresponding author on reasonable request.
